# Decreased Acetic Acid in the Stool of Preterm Infants Is Associated with an Increased Risk of Bronchopulmonary Dysplasia

**DOI:** 10.3390/nu14122412

**Published:** 2022-06-10

**Authors:** Lauren C. Frazer, William Yakah, Camilia R. Martin

**Affiliations:** 1 Department of Pediatrics, Division of Neonatal-Perinatal Medicine, University of North Carolina at Chapel Hill, Chapel Hill, NC 27599, USA; lauren_frazer@med.unc.edu; 2 Institute of Human Nutrition, Columbia University Medical Center, New York, NY 10032, USA; wy2381@cumc.columbia.edu; 3 Division of Newborn Medicine, New York-Presbyterian Hospital, Weill Cornell Medicine, New York, NY 10065, USA

**Keywords:** preterm infants, neonates, breast milk, stool, short-chain fatty acids, acetic acid, LC-MS/MS, bronchopulmonary dysplasia, retinopathy of prematurity, microbiome

## Abstract

Background: Short-chain fatty acids (SCFAs), microbial metabolites, have been minimally studied in neonatal pathophysiology but have been associated with disease outcomes in adults. The objective of this manuscript was to determine if SCFA levels in maternal breastmilk (BM) and stool from preterm neonates impacted the risk of neonatal morbidities. Methods: SCFA levels were quantified by liquid chromatography with tandem mass spectrometry on maternal BM and neonatal stool for preterm infants < 28 weeks’ gestation (N = 72) on postnatal days 14 and 28. SCFA levels in BM and stool of infants with and without bronchopulmonary disease (BPD) and retinopathy of prematurity (ROP) were compared. Logistic regression was applied to determine the association between stool acetic acid levels and disease. Results: Acetic, propionic, isobutyric, 2-methylbutyric, and isovaleric acid levels increased in BM and neonatal stool between days 14 and 28. Logistic regression demonstrated an inverse relationship between the quartile of fecal acetic acid level and the odds of BPD but not ROP on days 14 and 28. For each quartile increase in fecal acetic acid, the odds ratio (95% CI) of BPD was 0.41 (0.18, 0.83) for day 14 and 0.28 (0.09, 0.64) for day 28. Conclusions: Low acetic acid levels in the stool of preterm infants are associated with increased odds of BPD. These findings support a relationship between intestinal and pulmonary health in preterm infants.

## 1. Introduction

Preterm birth is associated with the development of numerous comorbidities, including bronchopulmonary dysplasia (BPD), retinopathy of prematurity (ROP), and necrotizing enterocolitis (NEC) [1]. The etiology of these conditions has not been completely elucidated but is multifactorial and associated with immature development, critical illness, iatrogenic injury, and deleterious inflammatory responses [2,3,4,5]. There is also a complex interplay between intestinal and overall health in preterm neonates. For example, severe NEC increases the risk of severe ROP and severe BPD [6]. Thus, further examination of markers of intestinal physiology in preterm infants may provide insight into new mechanisms underlying the development of a multitude of neonatal diseases. 

Short-chain fatty acids (SCFAs) are fatty acids that range from 1 to 6 carbons in length and are primarily produced in the colon by intestinal bacteria upon exposure to human milk oligosaccharides (HMOs) or indigestible dietary fiber [7]. Fecal SCFA levels thus reflect intestinal physiology as they are influenced by a combination of dietary intake and the composition of the microbiome. Despite the abundance of literature describing the role of SCFAs in systemic disease in adults, there is a paucity of research regarding the impact of SCFAs on the health of preterm neonates. In vitro studies using fetal intestinal cells and intestinal organoids demonstrated that the SCFAs acetate, propionate, and butyrate were anti-inflammatory [8]. In mice, acetate was shown to protect against hyperoxia-induced lung injury in a preclinical model of BPD [9]. A small study of preterm neonates determined that stool volatile organic compounds were significantly different between preterm neonates with severe BPD and controls [10]. Finally, a study of the early life microbiome and SCFA levels showed that an altered microbiome and decreased levels of fecal acetate were associated with an increased risk of asthma in children [11]. These findings support the notion of an inter-relationship between the health of the lung and gut. 

In this manuscript, we present the results of our single-center retrospective analysis of SCFA levels in the maternal breast milk and infant stool on days 14 and 28 after delivery for a cohort of neonates born at <28 weeks’ gestation. We examine SCFA levels over time and test the hypothesis that reduced fecal SCFA levels are associated with an increased risk of ROP and BPD. To our knowledge, this is the largest longitudinal study of SCFA levels in clinical samples from preterm infants with associated clinical outcomes data.

## 2. Materials and Methods

### 2.1. Cohort Selection and Study Design

This is a retrospective analysis of samples collected as a part of a prospective, non-interventional cohort study, which enrolled premature infants in the Infant Health Research Program between October 2009 and October 2012 at Beth Israel Deaconess Medical Center (BIDMC, Boston, MA, USA). The study was approved by the Institutional Review Board at BIDMC (Ref.: 2017P000632). Verbal informed consent was obtained from the parents of infants enrolled in the study, and all data were de-identified prior to analysis. The inclusion criteria were as follows: (1) gestational age of <28 weeks; (2) availability of maternal breastmilk and stool samples on day 14 and/or day 28 (±72 h); (3) receipt of maternal breastmilk feeds in the first 14 days; (4) absence of major congenital anomalies. A total of 72 infants met these inclusion criteria. A flow chart detailing infants in the entire cohort and those included in the analysis is provided in Supplementary Figure S1.

### 2.2. Maternal and Infant Data Collection

Maternal and infant clinical data were collected from the electronic medical records. Maternal data included age (years), mode of delivery (vaginal/C-section), gravida, multiparous (defined as parity more than 1), and presence of pregnancy-related morbidities such as preeclampsia, gestational diabetes, and pregnancy-induced hypertension. Infant data included gestational age (weeks), sex, birth weight (g) and the z-score (based on Fenton’s curve for preterm infants) [12], birth head circumference (cm), birth length (cm), Apgar score at one and five minutes infant Score of Neonatal Acute Physiology (SNAP, as an indicator of overall illness severity [13]), and growth velocity on days 14 and 28 (dichotomized as growth more or less than 10 g/kg/day). 

### 2.3. Neonatal Clinical Outcomes

BPD was defined as requiring supplemental oxygen at 36 weeks’ postmenstrual age. The presence of ROP was diagnosed by an ophthalmologist and defined as any level of ROP detected on a routine eye exam. Infants who were transferred to a different institution prior to reaching zone 3 and who did not have follow-up ROP data available were excluded from ROP outcomes analysis (4 infants). Neonatal clinical diagnoses included late-onset sepsis (defined as a positive blood culture any time after the third postnatal day), NEC (defined by the presence of pneumatosis intestinalis on abdominal radiograph), and overall mortality. Due to low numbers of infants with late-onset sepsis and/or NEC, only BPD and ROP were evaluated as primary outcomes. 

### 2.4. Breastmilk and Fecal Samples Collection

Feeding samples were collected as described previously [14]. At the end of each infant feeding, the bedside nurse placed an infusion tube with residual volume in a 4 °C refrigerator. Feeding samples were collected on days 14 and 28. After each 24-h collection period, samples were pooled, aliquoted, and stored in a −80 °C freezer until further analysis. Similarly, fecal samples were pooled from different diapers within the 24-h collection period, aliquoted, and stored in a −80 °C freezer until further analysis. A range of ±72 h for sample collection was permitted for each time point, given the variability in infant stooling patterns and breastmilk availability.

### 2.5. Short-Chain Fatty Acid Assay

Human breast milk and paired infant stool samples were analyzed for SCFAs by Metabolon (Durham, NC). These included acetic acid (C2), propionic acid (C3), isobutyric acid (C4), butyric acid (C4), 2-methyl-butyric acid (C5), isovaleric acid (C5), valeric acid (C5), and hexanoic acid (C6), which were quantified by liquid chromatography with tandem mass spectrometry (LC-MS/MS, Metabolon Methods TAM135 and TAM148). Human breast milk and paired infant stool samples were spiked with stable labeled internal standards and were subjected to protein precipitation with methanol. After centrifugation, an aliquot of the supernatant was derivatized. The reaction mixture was diluted for feces only and an aliquot was injected onto an Agilent 1290/AB Sciex QTrap 5500 LC-MS/MS system equipped with a C18 reversed-phase ultra-high-performance liquid chromatography (UHPLC) column. The mass spectrometer was operated in negative mode using electrospray ionization (ESI). The peak area of the individual analyte product ions was measured against the peak area of the product ions of the corresponding internal standards. Quantitation was performed using a weighted linear least squares regression analysis generated from fortified calibration standards prepared immediately prior to each run and raw data were collected and processed using AB SCIEX software Analyst 1.6.2 (SCIEX, MA). SCFA concentrations are given in ng/mL for breast milk and µg/g for stool (fresh weight).

### 2.6. Statistical Analyses

Results of univariate analysis of maternal and neonatal characteristics were expressed as mean ± standard deviation (SD) for continuous variables and proportion (%) for categorical variables. SCFAs with values below the limit of detection were replaced with ½ the minimum detected value as previously described [15]. Univariate analyses of SCFAs were performed to identify differences in SCFA levels between neonatal outcomes, BPD vs. no BPD, and ROP vs. no ROP. The *p*-values of univariate comparisons were adjusted for multiple comparisons. Next, we performed multivariable logistic regression analyses on significant SCFAs (classed into quartiles) to evaluate associations between quartile change in SCFA and risk of BPD or ROP, reported as odds ratios (95% CI) and *p*-values.

All analyses were performed using R software (version 3.5.2, R Core Team 2018a) within RStudio (Version 1.1.453, RStudio, Inc., Vienna, Austria) using tidyverse [16] and CompareGroups [17] packages. Statistical significance was defined as *p* < 0.05. 

## 3. Results

Maternal and neonatal characteristics of the 72 mother–infant dyads in this cohort are shown in Table 1. Overall, the average maternal age in this cohort was 31.2 ± 5.8 years, and 76.4% (55/72) of neonates were delivered by C-section. Maternal comorbidities included preeclampsia (12.5%), gestational diabetes mellitus (5.6%), and pregnancy-induced hypertension (9.7%). In the neonatal cohort, the average neonate had a gestational age of 26.3 ± 1.2 weeks, birthweight of 839 ± 187 g, and a 5-min Agar score of 7.2 ± 1.1. Of the 72 neonates, 45 (62.5%) developed BPD, 46 (63.9%) developed ROP, 12 (16.7%) had culture-positive sepsis, and 3 (4.2%) were diagnosed with NEC. 

**Figure 1:**
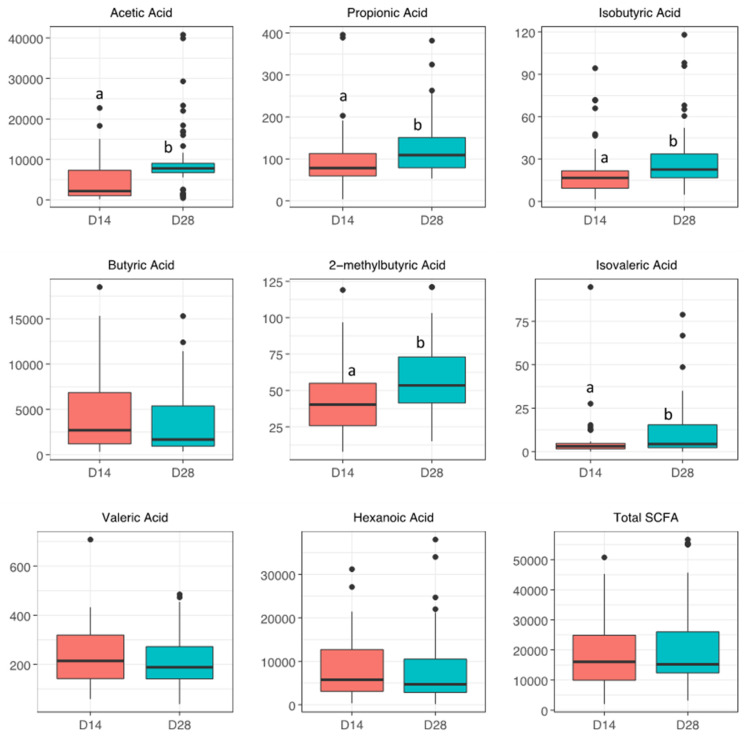
Short-chain fatty acid levels (ng/mL) in breastmilk samples on days 14 and 28. Boxplots show SCFA levels on days 14 and day 28. Wilcoxon tests were performed to test for statistical significance. Labeled points without a common letter are significantly different (*p* < 0.05).

**Figure 2:**
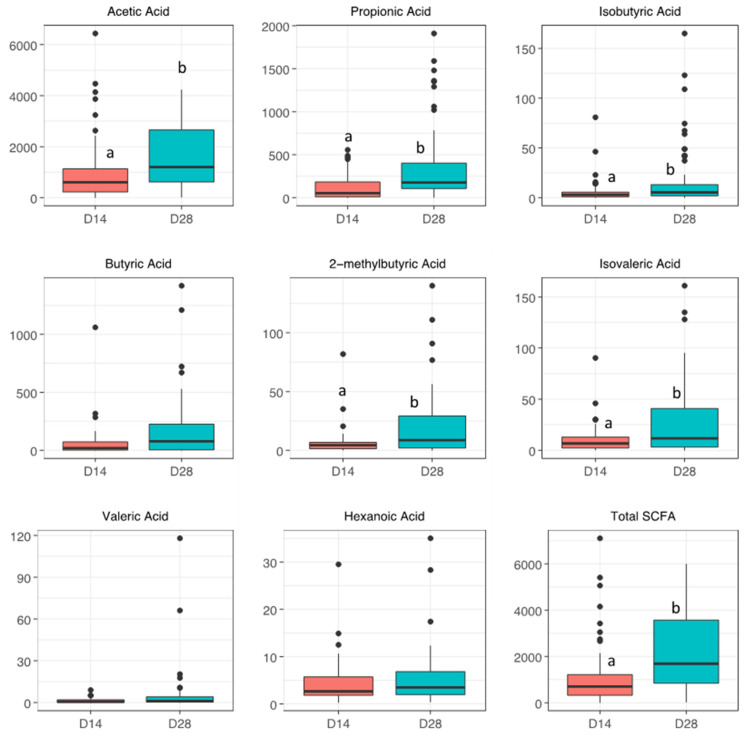
Short-chain fatty acid levels (ng/g) in infant stool samples on days 14 and 28. Boxplots show distribution of SCFAs on days 14 and day 28. Wilcoxon tests were performed to test for statistical significance. Labeled points without a common letter are significantly different (*p* < 0.05).

SCFA levels in maternal breastmilk and neonatal stool samples collected on day 14 and day 28 are shown in Figure 1 and Figure 2 and Supplementary Table S1. In breastmilk, acetic acid, the most abundant SCFA, was significantly increased on day 28 compared to day 14 (7800 ± 2315 vs. 2190 ± 6310 ng/mL, adjusted *p* < 0.001). Other less abundant SCFAs such as propionic acid, isobutyric acid, 2-methylbutyric acid, and isovaleric acid were all significantly increased in maternal breastmilk on day 28 compared to day 14 (Figure 1). Total SCFA levels in the breastmilk, however, did not significantly differ on day 28 compared to day 14 (*p* = 0.1). Similar to breastmilk, neonatal fecal acetic acid levels were significantly increased on day 28 compared to day 14 (1200 ± 2046 vs. 605 ± 909 μg/g, adjusted *p* = 0.002) alongside significant increases in propionic, isobutyric, 2-methylbutyric, and isovaleric acids (Figure 2). In contrast to breastmilk, total SCFA levels in the stool were significantly higher on day 28 compared to day 14 (*p* < 0.001).

Although there were no cohort differences in mothers of infants with BPD vs. no BPD, infants with BPD, compared to those without BPD, had lower gestational age (26.0 ± 1.2 vs. 26.9 ± 0.8 weeks, *p* < 0.001), birthweight (776 ± 168 vs. 966 ± 134 g, *p* < 0.001), and 5-min Apgar (6.9 ± 1.2 vs. 7.7 ± 0.9, *p* = 0.002) (Table 2). Similarly, infants with ROP, compared to those with no ROP, were of a lower gestational age (26.0 ± 1.2 vs. 26.9 ± 1.0 weeks, *p* = 0.001), and birthweight (793 ± 180 vs. 932 ± 140 g, *p* = 0.001), while 5-min Apgar scores were comparable (7.2 ± 1.0 vs. 7.1 ± 1.5, *p* = 0.9) (Table 2). Total days of exposure to antibiotics in the first 14 and 28 days were higher in infants with BPD *(p* < 0.05) but comparable for infants with or without ROP. The score of neonatal acute physiology (SNAP), a marker of disease severity, was significantly higher in infants with BPD compared to no BPD (15.7 ± 8.5 vs. 8.8 ± 7.8, *p* = 0.001) and trending towards significance for infants with ROP compared to no ROP (14.9 ± 8.7 vs. 11.1 ± 8.0, *p* = 0.08).

Differences in breastmilk and fecal SCFA levels between neonatal outcomes, BPD vs no BPD, and ROP vs no ROP, are characterized in Table 3 and Table 4, respectively. In maternal breastmilk samples on days 14 and 28, no SCFAs were significantly different for infants with BPD compared to no BPD, or ROP compared to no ROP, after adjusting for multiple comparisons (Table 3); however, in infants’ stool samples on day 14, acetic acid approached significance for infants with BPD compared to no BPD (446 ± 564 vs. 1000 ± 712 μg/g, adjusted *p* = 0.05) (Table 4). This difference was not observed in infants with ROP compared to no ROP (595 ± 813 vs. 918 ± 1097 μg/g, adjusted *p* = 0.3). Total SCFAs on day 14 also approached significance for infants with BPD compared to infants without BPD (482 ± 812 vs. 1051 ± 896 μg/g, adjusted *p* = 0.05). Similarly, on day 28, infants with BPD compared to no BPD had a trend toward decreased levels of total SCFA (1252 ± 1389 vs. 2749 ± 2312 μg/g, adjusted *p* = 0.06), although these levels were comparable in ROP after adjusting for multiple comparisons. 

To further examine the association between changes in acetic acid levels and risk of developing BPD or ROP, we performed a multivariable binary logistic regression analysis to determine the relationship between disease vs no disease, for every quartile increase in fecal acetic acid levels as shown in Table 5. SCFA levels were expressed as quartiles to assess both high and low concentrations on the impact of disease, to account for the lack of known normal values of SCFAs longitudinally in preterm infants, and to account for the non-normal distribution of these metabolites [18]. On day 14, per quartile increase in acetic acid was associated with a decreased likelihood of developing BPD (odds ratio, 0.41; 95% CI 0.18–0.83; *p* = 0.02), with the highest quartile (compared to the lowest quartile) conferring the least likelihood of developing BPD (Table 5). Similarly, on day 28, per quartile increase in acetic acid was also associated with a decreased likelihood of developing BPD (odds ratio, 0.28; 95% CI 0.09–0.64; *p* = 0.009). No significant associations were observed between acetic acid levels and risk of developing ROP on day 14 or 28.

## 4. Discussion

In this manuscript, we present the results of a comprehensive longitudinal analysis of SCFA levels in both maternal breastmilk and neonatal stool for a cohort of infants born at <28 weeks’ gestation. This is the largest study to date of SCFA levels in preterm neonates and the only study to analyze SCFAs in both maternal breastmilk and neonatal stool of maternal–infant dyads. In addition, we present novel data on breastmilk and stool SCFA levels over early life and in association with the risk of BPD and ROP.

In this study, we show that levels of acetic, propionic, isobutyric, 2-methylbutyric, and isovaleric acid increase in maternal breastmilk and neonatal stool between days 14 and 28 after delivery. This is in accordance with a previous study of 51 infants born at <32 weeks’ gestation that found an increase in stool SCFAs associated with the initiation of enteral feeding and advancing infant age [19]. SCFAs are synthesized by specific strains of intestinal bacteria upon encountering indigestible dietary components such as human milk oligosaccharides (HMOs) [20,21]. Thus, this temporal increase in SCFAs is likely related to the increased bacterial abundance that occurs in the gut of preterm infants over the first 40 days after delivery [22] as well as by increased substrate provided to SCFA producing bacteria in the context of increasing enteral feeding volume. In addition, it is likely that changes in breastmilk SCFAs are reflective of maternal circulating levels, although it is possible there is a contribution from local production by an interaction of the breastmilk microbiome and HMOs. A previous study of preterm infants found significantly increased acetate and propionate in the stool of infants receiving expressed breastmilk compared to those receiving preterm formula [19]. Thus, SCFA levels provide a functional readout of the activity and composition of the neonatal microbiome as well as the nutritional intake of the neonate.

Our data demonstrate that acetic acid levels in the stool of preterm infants on day 14 and day 28 are inversely associated with the risk of BPD using a multivariable logistic regression analysis. In contrast, there was no association between stool levels of any of the SCFAs and the risk of ROP. The specificity for BPD may reflect a link between intestinal health and pulmonary outcomes in preterm neonates. This theory is supported by both preclinical and clinical data. A study in mice found a direct association between dietary fiber intake, gut microbiota, SCFA production, and the development of allergic airway disease [23]. Another study in mice found that increased maternal intake of dietary fiber or maternal exposure to acetic acid led to reduced allergic airway disease in offspring via direct regulation of gene transcription in the fetal lung and augmentation of regulatory T cell function [24]. In addition, decreased levels of fecal acetate have been associated with an increased risk of asthma in children [11]. Maternal prebiotic supplementation during pregnancy is currently under investigation as an immunomodulatory prevention strategy for atopic disease in children in the PREGRALL [25] and SYMBA [26] studies. SCFAs have a multitude of immunoregulatory functions [27], and it is possible that acetic acid acts on intestinal immune cells, which then circulate and influence pulmonary outcomes, or that acetic acid is absorbed into the systemic circulation and directly modulates immune cells in the lungs. 

The neonates that developed BPD in this study were of a significantly younger gestational age and smaller size at birth, in addition to having a higher SNAP score compared to infants who did not develop BPD. We corrected for these variables in our multivariable logistic regression model of acetic acid levels and the risk of BPD, and a strong inverse relationship between acetic acid levels and BPD risk remained. In addition, the infants who developed ROP were also younger, smaller, and sicker than their non-ROP counterparts, but ROP did not have a significant association with SCFA levels. This is possibly because ROP was defined as “any level of ROP”, and we may have missed an association with infants with more severe disease. It is also possible that there is a specific interaction between pulmonary and intestinal health mediated by acetic acid, as previously discussed. 

The limitations of this study include that these samples were collected from 2009–2012 at a single center. Clinical practices have changed since that time with increased antibiotic stewardship and earlier initiation of feeds, though we did adjust for days of antibiotic use. In addition, stool SCFA levels may not necessarily reflect intestinal or systemic SCFA levels as >95% of SCFAs are taken up by colonocytes and not excreted in the stool [28]. Finally, this is a correlative, hypothesis-generating study. We cannot determine causation with these data, and that should be explored with mechanistic basic science studies, which could then lay the foundation for a larger randomized controlled clinical trial. 

SCFA levels represent a unique tool for analyzing the metabolic activity of an infant’s microbiome. The microbiome of preterm infants diverges from that of a healthy term infant and is characterized by a preponderance of pathogenic bacteria [22]. Although probiotics are an appealing idea for the modulation of the preterm infant microbiome, the American Academy of Pediatrics currently recommends against probiotic use for preterm neonates given unclear benefits, the lack of clinical-grade product, and safety concerns [29]. Further high-quality studies into probiotics and possibly a combination of prebiotics and probiotics are needed before this strategy can be adapted into clinical practice [30].

Our findings of a possible role for acetic acid in preventing BPD provide further support for neonatologists to prioritize the intestinal health of preterm neonates. Clinical approaches that aim to protect and restore the microbiome of preterm neonates via early breastmilk feedings and reduced antibiotic exposure may prevent both intestinal and pulmonary disease. 

## Figures and Tables

**Table 1:** Maternal and neonatal characteristics of the study cohort ^1^.

N = 72	Mean ± SD	Range
Maternal characteristics		
Maternal age (years)	31.2 ± 5.84	18.0–49.0
Race, White	40 (55.6%)	-
Delivery, c-section	55 (76.4%)	-
Gravida (≥2)	53 (73.6%)	-
Multiparous	8 (11.1%)	-
Preeclampsia	9 (12.5%)	-
Gestational diabetes mellitus	4 (5.6%)	-
Pregnancy-induced hypertension	7 (9.7%)	-
Neonatal characteristics		
Gestational age (weeks)	26.3 ± 1.19	23.4–27.9
Sex, female	36 (50.0%)	-
Birthweight (g)	839 ± 187	350–1160
Birthweight z-score	−0.10 ± 0.90	−2.89–1.61
Birth head circumference (cm)	24.0 ± 2.04	19.5–34.5
Birth length (cm)	34.3 ± 3.18	25.5–45.5
Apgar (1min)	5.19 ± 2.10	1.0–8.0
Apgar (5min)	7.18 ± 1.14	3.0–9.0
Day 14 GV > 10 g/k/d	11 (15.3%)	-
Day 28 GV > 10 g/k/d	35 (48.6%)	-
Days of abx in first 14 days	6.28 ± 4.11	2.0–14.0
Days of abx in first 28 days	9.12 ± 7.15	2.0–28.0
SNAP Score	13.9 ± 9.15	0.0–36.0
BPD	45 (62.5%)	-
ROP	46 (63.9%)	-
NEC	3 (4.2%)	-
Sepsis (blood culture)	12 (16.7%)	-
Mortality	4 (5.6%)	-

^1^ Data are presented as mean ± SD and n (percentages) for quantitative and qualitative variables, respectively. Abx, antibiotics; BPD, bronchopulmonary dysplasia; GV, growth velocity; NEC, necrotizing enterocolitis; ROP, retinopathy of prematurity; SNAP, score of neonatal acute physiology.

**Table 2:** Maternal and neonatal characteristics of the study cohort according to neonatal disease outcomes ^1^.

	BPD			ROP		
	No	Yes	*p*	No	Yes	*p*
N	27	45		22	46	
Maternal characteristics						
Maternal age (years)	32.3 ± 6.61	30.7 ± 5.68	0.3	32.0 ± 5.80	31.1 ± 6.33	0.6
Race, White	17 (63.0%)	22 (48.9%)	0.4	16 (72.7%)	21 (45.7%)	0.07
Delivery			0.3			0.09
Vaginal	10 (37.0%)	10 (22.2%)		10 (45.5%)	10 (21.7%)	
C-section	17 (63.0%)	35 (77.8%)		12 (54.5%)	36 (78.3%)	
Gravida (≥2)	17 (63.0%)	32 (71.1%)	0.7	14 (63.6%)	32 (69.6%)	0.8
Multiparous	2 (7.41%)	5 (11.1%)	0.7	0 (0.00%)	6 (13.0%)	0.2
Preeclampsia	1 (3.70%)	7 (15.6%)	0.2	1 (4.55%)	7 (15.2%)	0.3
Gestational diabetes mellitus	3 (11.1%)	1 (2.22%)	0.2	3 (13.6%)	1 (2.17%)	0.1
Pregnancy-induced hypertension	3 (11.1%)	3 (6.67%)	0.7	2 (9.09%)	4 (8.70%)	1.0
Neonatal characteristics						
Gestational age (weeks)	26.9 ± 0.81	26.0 ± 1.24	**<0.001**	26.9 ± 0.96	26.0 ± 1.18	**0.001**
Sex, female	9 (33.3%)	24 (53.3%)	0.2	6 (27.3%)	26 (56.5%)	**0.05**
Birthweight (g)	966 ± 134	776 ± 168	**<0.001**	932 ± 140	793 ± 180	**0.001**
Birthweight z-score	0.30 ± 0.62	−0.30 ± 0.92	**0.002**	0.09 ± 0.56	−0.18 ± 0.98	0.2
Birth head circumference (cm)	25.4 ± 2.23	23.3 ± 1.33	**<0.001**	24.8 ± 1.44	23.7 ± 2.21	**0.02**
Birth length (cm)	35.8 ± 1.81	33.4 ± 2.72	**<0.001**	36.3 ± 2.05	33.3 ± 2.57	**<0.001**
Apgar (1 min)	5.96 ± 1.81	4.91 ± 2.18	**0.03**	5.73 ± 2.14	5.07 ± 2.13	0.2
Apgar (5 min)	7.70 ± 0.87	6.93 ± 1.19	**0.002**	7.14 ± 1.46	7.20 ± 0.98	0.9
Day 14 GV > 10 g/k/d	2 (7.69%)	7 (16.7%)	0.5	3 (14.3%)	6 (14.0%)	1.0
Day 14 BM volume (ml/kg)	551 ± 364	320 ± 273	**0.007**	454 ± 331	369 ± 329	0.3
Days of abx use in first 14 days	4.70 ± 3.17	6.93 ± 4.23	**0.01**	5.41 ± 4.12	6.78 ± 3.88	0.2
Days of abx use in first 28 days	6.37 ± 6.35	9.98 ± 6.67	**0.03**	7.64 ± 7.59	9.63 ± 6.31	0.3
SNAP Score	8.78 ± 7.80	15.7 ± 8.45	**0.001**	11.1 ± 8.01	14.9 ± 8.74	0.08
PDA closure (indomethacin)	17 (63.0%)	33 (73.3%)	0.5	13 (59.1%)	34 (73.9%)	0.3
NEC	0 (0.00%)	3 (6.98%)	0.3	1 (4.76%)	2 (4.55%)	1.0
Sepsis (blood culture)	3 (11.5%)	8 (18.2%)	0.5	3 (14.3%)	8 (17.8%)	1.0
Mortality	0 (0.00%)	2 (4.76%)	0.5	1 (4.76%)	1 (2.33%)	1.0

^1^ Data are presented as mean ± SD and percentages (*n*) for quantitative and qualitative variables, respectively. BM, breastmilk; BPD, bronchopulmonary dysplasia; GV, growth velocity; NEC, necrotizing enterocolitis; ROP, retinopathy of prematurity; SNAP, score of neonatal acute physiology. Bold font is used to highlight significant differences.

**Table 3:** Comparison of short-chain fatty acid levels in maternal breastmilk on day 14 and day 28 after delivery based on neonatal comorbidities ^1^.

SCFA (ng/mL)	No BPD	BPD	*p*	*p* (Adj)	No ROP	ROP	*p*	*p* (Adj)
BM Day 14								
N	26	42			21	43		
Acetic	3590 [1080, 7185]	1730 [810, 7615]	0.7	0.8	5020 [891, 8280]	1710 [1010, 7055]	0.2	0.4
Propionic	73.8 [57.5, 110]	87.3 [63.6, 120]	0.4	0.8	82.8 [58.8, 156]	76.1 [58.1, 108]	0.7	0.8
Isobutyric	18.0 [11.6, 21.8]	12.6 [8.14, 23.1]	0.2	0.8	16.8 [9.25, 23.9]	16.6 [8.67, 23.0]	0.9	0.9
Butyric	2390 [1198, 5615]	3345 [1272, 7012]	0.7	0.8	2130 [982, 3970]	4390 [1240, 7290]	0.1	0.4
2-methylbutyric	**47.9 [39.3, 61.6]**	**32.8 [22.8, 49.7]**	**0.01**	0.1	43.2 [35.3, 61.9]	32.9 [23.9, 51.8]	0.2	0.4
Isovaleric	2.88 [1.52, 3.81]	3.10 [1.76, 4.96]	0.6	0.8	1.98 [1.39, 3.78]	3.22 [1.47, 4.70]	0.6	0.7
Valeric	180 [140, 313]	238 [172, 328]	0.4	0.8	183 [108, 260]	232 [158, 336]	0.05	0.4
Hexanoic	6430 [3500, 13400]	7595 [2640, 12175]	0.5	0.8	4660 [1820, 8680]	9020 [3300, 14100]	0.1	0.4
Total SCFA	14857 [9948, 25013]	16797 [10451, 24277]	0.9	0.9	12617 [9566, 22302]	17456 [10823, 26175]	0.4	0.5
BM Day 28								
N	23	41			18	43		
Acetic	7850 [6605, 9530]	7670 [6900, 9150]	1.0	1.0	**8415 [7168, 18050]**	**7500 [6215, 8830]**	**0.03**	0.1
Propionic	111 [74.7, 158]	108 [84.8, 139]	1.0	1.0	126 [87.6, 166]	103 [76.0, 126]	0.08	0.2
Isobutyric	20.5 [14.3, 33.6]	22.7 [17.5, 33.7]	0.6	1.0	25.8 [18.7, 36.8]	21.2 [14.4, 32.9]	0.2	0.3
Butyric	1790 [1125, 5970]	1670 [892, 3590]	0.6	1.0	1275 [930, 5450]	1890 [887, 5540]	0.8	0.9
2-methylbutyric	54.1 [42.8, 73.5]	51.8 [40.7, 71.2]	0.7	1.0	64.2 [46.6, 73.2]	44.3 [38.1, 65.9]	0.07	0.2
Isovaleric	3.96 [2.27, 19.4]	4.27 [1.41, 9.68]	0.8	1.0	**18.9 [4.13, 25.7]**	**3.49 [1.32, 7.94]**	**0.02**	0.1
Valeric	214 [140, 242]	185 [155, 298]	0.4	1.0	202 [156, 229]	184 [140, 294]	0.9	0.9
Hexanoic	4890 [2645, 12450]	4590 [3310, 9760]	1.0	1.0	4210 [2435, 11590]	4890 [3475, 10500]	0.4	0.6
Total SCFA	15597 [12498, 29200]	15220 [12091, 25028]	0.7	1.0	19381 [12416, 39166]	14948 [11843, 25907]	0.3	0.5

^1^ Short-chain fatty acid levels are reported as median (Q1, Q3). Wilcoxon tests were performed to determine statistical significance, and false discovery rate (FDR) *p*-value adjustments are performed to correct for multiple comparisons (*p* (Adj)). Bold font is used to highlight significant differences.

**Table 4:** Short-chain fatty acid levels in neonatal stool samples on day 14 and day 28 after delivery based on neonatal comorbidities ^1^.

SCFA (ng/mL)	No BPD	BPD	*p*	*p* (Adj)	No ROP	ROP	*p*	*p* (Adj)
Stool Day 14								
N	26	37			20	39		
Acetic Acid	**1000 [656, 1368]**	**446 [215, 779]**	**0.01**	0.05	918 [415, 1512]	595 [212, 1025]	0.09	0.4
Propionic Acid	72.2 [11.2, 167]	22.9 [12.0, 99.1]	0.5	0.5	41.1 [14.4, 120]	52.2 [11.0, 121]	0.9	0.9
Isobutyric Acid	3.03 [1.44, 12.1]	3.29 [1.03, 5.39]	0.2	0.4	3.38 [1.59, 5.78]	3.29 [1.10, 5.65]	0.7	0.9
Butyric Acid	27.2 [18.4, 151]	4.43 [1.82, 65.5]	0.4	0.5	37.7 [20.3, 202]	18.4 [2.14, 92.3]	0.3	0.6
2-methylbutyric Acid	4.78 [2.14, 7.97]	2.91 [1.27, 5.82]	0.2	0.4	3.68 [1.85, 5.04]	4.78 [1.22, 7.73]	0.9	0.9
Isovaleric Acid	9.69 [2.96, 13.0]	7.43 [2.44, 13.0]	0.5	0.5	6.96 [3.38, 13.0]	7.15 [2.54, 13.1]	0.8	0.9
Valeric Acid	0.87 [0.19, 2.80]	0.54 [0.13, 0.91]	0.2	0.4	0.19 [0.14, 2.30]	0.62 [0.19, 1.38]	0.7	0.9
Hexanoic Acid	2.15 [1.71, 5.20]	2.86 [1.95, 5.72]	0.5	0.5	**2.02 [1.54, 2.22]**	**3.84 [2.09, 6.28]**	**0.01**	0.05
Total SCFA	**1051 [726, 1622]**	**482 [235, 1047]**	**0.01**	0.05	977 [498, 1791]	658 [271, 1095]	0.1	0.4
Stool Day 28								
	24	41			21	40		
Acetic Acid	**1790 [1115, 3105]**	**905 [614, 1910]**	**0.03**	0.1	1820 [809, 3280]	1055 [618, 1932]	0.1	0.3
Propionic Acid	**395 [126, 594]**	**142 [47.1, 318]**	**0.02**	0.08	307 [131, 471]	136 [45.3, 376]	0.1	0.3
Isobutyric Acid	6.29 [2.96, 19.3]	5.15 [1.59, 13.8]	0.38	0.4	6.08 [2.96, 29.4]	5.15 [1.92, 11.1]	0.5	0.6
Butyric Acid	114 [45.2, 264]	11.8 [1.96, 160]	0.06	0.1	114 [18.1, 255]	57.8 [2.68, 177]	0.2	0.4
2-methylbutyric Acid	13.8 [4.04, 34.5]	5.93 [1.33, 16.4]	0.09	0.1	10.8 [3.94, 24.3]	5.93 [1.73, 20.3]	0.3	0.4
Isovaleric Acid	29.1 [5.66, 58.0]	10.6 [2.90, 36.2]	0.10	0.1	19.8 [5.66, 40.1]	10.6 [2.37, 35.5]	0.3	0.4
Valeric Acid	1.07 [0.36, 5.11]	0.65 [0.18, 3.16]	0.29	0.4	1.16 [0.20, 8.71]	0.94 [0.30, 3.17]	0.5	0.6
Hexanoic Acid	3.41 [2.12, 7.17]	3.81 [2.40, 7.04]	0.58	0.6	3.50 [2.25, 5.60]	3.96 [2.42, 7.27]	0.6	0.6
Total SCFA	**2749 [1594, 3906]**	**1252 [756, 2145]**	**0.01**	0.06	2567 [1165, 4245]	1334 [789, 2320]	0.08	0.3

^1^ Short-chain fatty acid levels are reported as median (Q1, Q3). Wilcoxon tests were performed to determine statistical significance, and false discovery rate (FDR) *p*-value adjustments are performed to correct for multiple comparisons (*p* (Adj)). Bold font is used to highlight significant differences.

**Table 5:** Association of acetic acid, as quartiles, with risk of neonatal disease, BPD, and ROP ^1^.

	BPD ^1^ OR (95% CI)	*p*-Value	ROP ^1^ OR (95% CI)	*p*-Value
Day 14				
Acetic acid (per quartile increase)	**0.41 [0.18, 0.83]**	**0.02**	0.66 [0.35, 1.17]	0.2
Q2 vs. Q1	1.51 [0.15, 1.74]	0.7	0.42 [0.06, 2.67]	0.4
Q3 vs. Q1	0.18 [0.02, 1.45]	0.1	1.62 [0.21, 13.4]	0.6
Q4 vs. Q1	0.11 [0.008, 0.98]	0.06	0.18 [0.02, 1.17]	0.08
Day 28				
Acetic acid (per quartile increase)	**0.28 [0.09, 0.64]**	**0.009**	0.62 [0.32, 1.11]	0.1
Q2 vs. Q1	0.28 [0.01,3.86]	0.4	0.61 [0.08, 4.51]	0.6
Q3 vs. Q1	0.06 [0.003, 0.78]	0.05	0.93 [0.11, 7.21]	0.9
Q4 vs. Q1	**0.02 [0.001, 0.31]**	**0.01**	0.20 [0.03, 1.28]	0.1

^1^ Logistic regression models for both BPD and ROP were adjusted for gestational age, birthweight z-score, score of neonatal acute physiology (SNAP), days of antibiotics use either in the first 14 or 28 days, and breastmilk volume (mL/kg). Odds ratios are referenced to no disease group. Bold font is used to highlight significant differences.

## Data Availability

Data are available by request to the corresponding author.

## Supplementary Materials

The following supporting information can be downloaded at: https://www.mdpi.com/article/10.3390/nu14122412/s1, Figure S1: Characteristics of infants in the entire cohort and those included in this study. Table S1: Levels of breastmilk and stool short-chain fatty acids (ng/mL) on day 14 and 28.
