# Supplementary material for: Decreased Acetic Acid in the Stool of Preterm Infants Is Associated with an Increased Risk of Bronchopulmonary Dysplasia
Source: Nutrients. 2022 Jun 10;14(12):2412. doi: 10.3390/nu14122412 (PMC9230097; doi:10.3390/nu14122412)
Supplement: Supplementary file 1 [file nutrients-14-02412-s001.zip › nutrients-1738678-supplementary.pdf]

Supplementary Materials

Figure S1. Characteristics of infants in the entire cohort and those included in this study.

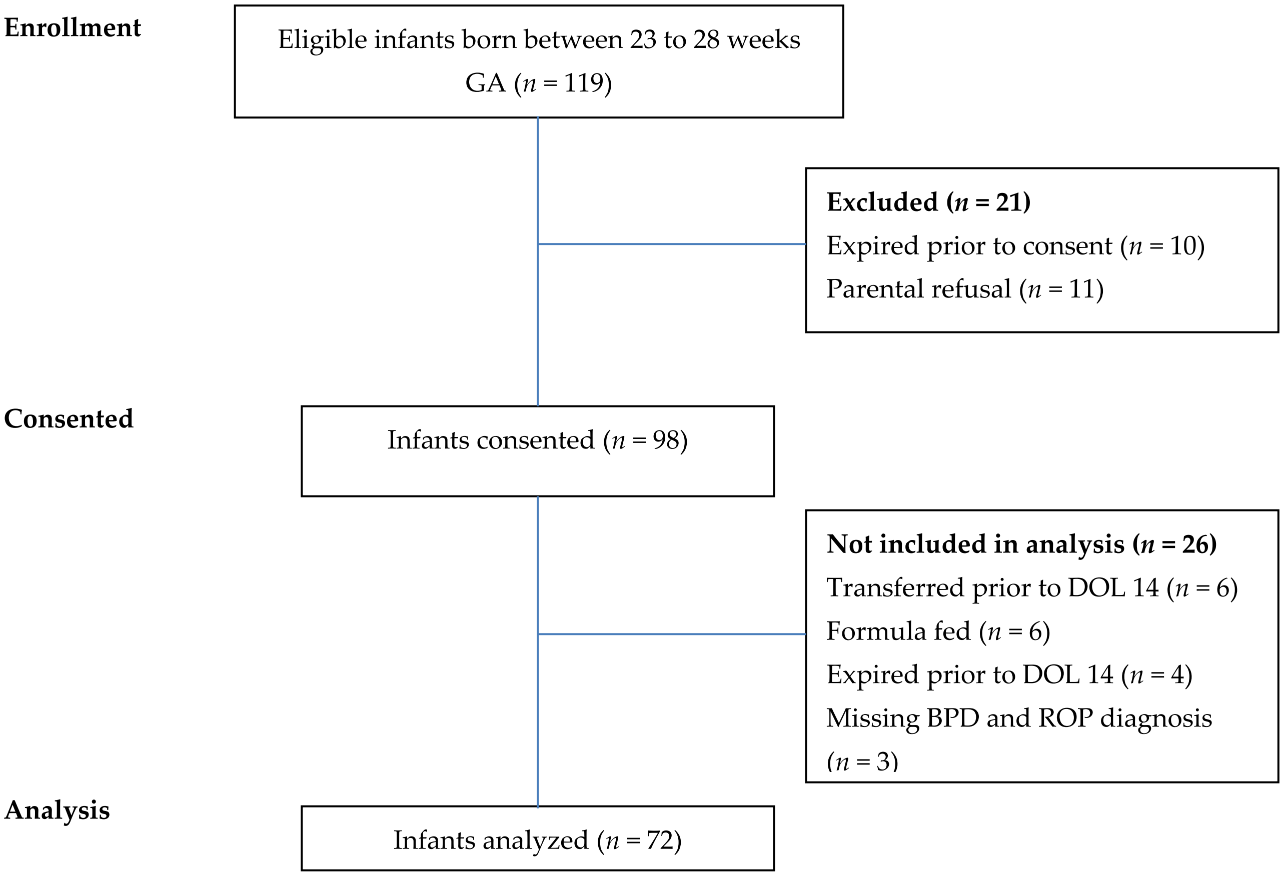

Table S1. Levels of breastmilk and stool short chain fatty acids (ng/mL) at day 14 and 28.[1]

SCFA	Day 14	Day 28	*P*	*P* (Adj)
**Breastmilk**				
N	68	64		
Acetic Acid	2190 [1020;7330]	7800 [6745;9060]	**<0.001**	**<0.001**
Propionic Acid	78.4 [59.3;113]	109 [78.8;151]	**<0.001**	**<0.001**
Isobutyric Acid	16.6 [9.29;21.7]	22.6 [16.7;33.5]	**<0.001**	**<0.001**
Butyric Acid	2700 [1190;6870]	1670 [935;5395]	0.07	0.1
2-methylbutyric Acid	40.3 [25.8;55.0]	53.5 [41.4;73.0]	**<0.001**	**<0.001**
Isovaleric Acid	3.03 [1.52;4.71]	4.27 [2.20;15.4]	**0.04**	0.08
Valeric Acid	214 [142;319]	188 [141;272]	0.4	0.4
Hexanoic Acid	5750 [3120;12700]	4700 [2845;10500]	0.6	0.6
Total SCFA	16061 [9936;24884]	15220 [12368;26012]	0.1	0.2
**Stool**				
N	63	65		
Acetic Acid	605 [221;1130]	1200 [614;2660]	**0.001**	**0.002**
Propionic Acid	52.5 [11.0;183]	177 [108;401]	**<0.001**	**0.002**
Isobutyric Acid	2.98 [1.06;5.61]	5.42 [1.92;13.2]	**0.007**	**0.01**
Butyric Acid	18.4 [1.59;71.6]	76.7 [2.97;224]	0.09	0.1
2-methylbutyric Acid	4.39 [1.34;6.81]	8.62 [1.98;29.3]	**0.006**	**0.01**
Isovaleric Acid	6.62 [2.23;12.9]	11.7 [3.09;40.8]	**0.02**	**0.03**
Valeric Acid	0.62 [0.15;1.91]	0.94 [0.32;4.15]	0.1	0.2
Hexanoic Acid	2.66 [1.84;5.72]	3.50 [1.97;6.82]	0.2	0.1
Total SCFA	701 [323;1217]	1687 [847;3569]	**<0.001**	**<0.001**

[1]Short chain fatty acid levels are reported as median (Q1, Q3). Wilcoxon tests were performed to determine statistical significance, and False Discovery Rate (FDR) *P*-value adjustments were performed to correct for multiple comparisons. Bold font is used to highlight significant differences.
